# Comprehensive Nutrition Review of Grain-Based Muesli Bars in Australia: An Audit of Supermarket Products

**DOI:** 10.3390/foods8090370

**Published:** 2019-08-28

**Authors:** Felicity Curtain, Sara Grafenauer

**Affiliations:** 1 Grains & Legumes Nutrition Council, Mount Street, North Sydney 2060, Australia; 2 School of Medicine, University of Wollongong, Northfields Avenue, Wollongong 2522, Australia

**Keywords:** muesli bars, grains, whole grain, dietary fibre, snack foods, nutrition

## Abstract

Muesli bars are consumed by 16% of children, and 7.5% of adults, and are classified as discretionary in Australian Dietary Guidelines, containing “higher fat and added sugars” compared with core food choices. This study aimed to provide a nutritional overview of grain-based muesli bars, comparing data from 2019 with 2015. An audit of muesli bars, grain-based bars, and oat slices was undertaken in January 2019 (excluding fruit, nut, nutritional supplement, and breakfast bars) from the four major supermarkets in metropolitan Sydney. Mean and standard deviation was calculated for all nutrients on-pack, including whole grain per serve and per 100g. Health Star Rating (HSR) was calculated if not included on-pack. Of all bars (*n* = 165), 63% were ≤ 600 kJ (268–1958 kJ), 12% were low in saturated fat, 56% were a source of dietary fibre, and none were low in sugar. Two-thirds (66%) were whole grain (≥8 g/serve), with an average of 10 g/serve, 16% of the 48 g Daily Target Intake. HSR featured on 63% of bars (average 3.2), with an overall HSR of 2.7. Compared to 2015, mean sugars declined (26.6 g to 23.7 g/100 g; *p* < 0.001), and 31% more bars were whole grain (109 up from 60 bars). Although categorised as discretionary, there were significant nutrient differences across grain-based muesli bars. Clearer classification within policy initiatives, including HSR, may assist consumers in choosing products high in whole grain and fibre at the supermarket shelf.

## 1. Introduction

‘Muesli bar’ is a generic term that refers to baked or cold-formed cereal-based snack bars, and may contain other ingredients such as fruit, nuts, seeds, chocolate, yoghurt, and a variety of other fillings and/or toppings [1]. They are a popular food in Australia, with consumption per capita considered the third highest worldwide, behind Canada and the USA [2]. An estimated 7.5% of Australian adults ate muesli bars the day prior to the 2011–12 Australian Health Survey, with consumption more common in younger age groups (16% of 4–13 year olds, compared to 12.8% of 14–18 year olds, and less than 8% of those aged 19–50 years) [3]. Their popularity with children was noted in a 2005 paper reviewing the lunchbox content of Australian school children, which found an estimated 41.8% of lunchboxes included a muesli/fruit bar, though this also included non-grain-based bars, excluded from this research [4].

Data from the 2011–12 Australian Health Survey found muesli bars contributed overall less than 1% of total energy, protein, fats, sugars, and dietary fibre to Australians aged 2 years and older [5]. However for females aged between 2–18 years, these figures were slightly higher; 1.1% energy, 1.2% total sugars, and 1.5% dietary fibre, and for males 2–18 years; 1.2% energy, 1% saturated fat, 1.4% total sugars, and 1.6% dietary fibre [5]. There is a lack of consensus on what constitutes a ‘snack food’, with definitions ranging from foods consumed between main meals or at specific times of day, food-type, or participant-described. Based on ‘time of day’ consumption, bars can be considered a snack food, generally eaten between main meals, and snacking of this kind has been linked with concern around increased risk of obesity and related chronic disease [6], though importantly, these health outcomes are multifactorial, with food choice and energy balance key in determining whether snacking is a healthful or harmful food behaviour [7,8].

Between 1995 and 2012, the prevalence and frequency of children snacking (defined as a single eating occasion between main meals) rose in Australia, with more than double the number of children snacking four or more times per day in 2012 [9]. Subsequently, the contribution of snacks to total energy intake significantly increased, from 24–30.5%. Foods consumed as snacks were a mix of traditional ‘snack’ foods such as sweet biscuits, cakes, fresh fruit, and ‘meal’ foods, such as bread and milk. Fruit and vegetable juice was the top contributor to energy from snacks in 1995, but did not appear in 2012, with pome fruit moving up as the top contributor. Muesli bars did not feature in the top snacks in 1995, but were number seven in 2007, and number nine in 2012, where they contributed an estimated 12.5% of total energy to snacks [9]. In Australian adults, cakes, muffins, scones, breads, and dairy milk were the three greatest contributors to energy from snacks, with 22% of total energy derived from snacking occasions [10]. While no data has reviewed changes in snacking habits among Australian adults, steady increases from 1977–2006 amongst adults in the USA mirror Australian children’s results, contributing more kilojoules, mainly from discretionary foods like desserts, sugar sweetened beverages, and salty snacks [11].

The popularity of muesli bars, and increasing levels of consumption [9] have attracted attention from public health groups, government, and the media, not least since they are considered a ‘discretionary’ food in the Australian Dietary Guidelines, where their consumption is discouraged based on having “higher fat and added sugars” [12]. Importantly, they are not depicted in the accompanying Australian Guide to Healthy eating, which visually represents core and discretionary foods. Instead, muesli bars are listed in the longer form supplementary text, and are therefore hidden from view, so it is unclear how well understood their classification as discretionary is among consumers. Similarly, the New Zealand Eating and Activity Guidelines present muesli bars as an example of a ‘highly processed’ food that may be refined and contain added saturated fat, sugar, and salt [13], and the United Kingdom’s Eat Well Guide cautions that cereal bars may have high levels of added sugars [14].

In 2018, proposed sugar reformulation targets for muesli bars were developed by The Healthy Food Partnership, an initiative established by the Australian Government in 2015, which aims to improve public health nutrition through several policy areas, including food reformulation [1]. Their inclusion was noteworthy, as they did not comply with the initial criteria (contributing significantly (≥1%) to sodium, sugars, and/or saturated fat in the Australian population’s intake), instead being included based on their high level of consumption among children [1]. The proposed targets call for a “10% reduction in sugar across defined products containing over 28 g sugar/100 g, and a reduction in sugar to 25 g/100 g for products between 25–28 g sugar/100 g by the end of 2022”. It is important to recognise that many companies have their own nutrition policies and commitments, as outlined in a 2018 Australian report, which found 16 of the 19 food companies surveyed included nutrition in their corporate strategy and had a commitment to product reformulation, while 11 out of 19 had committed to implementing the voluntary Health Star Rating (HSR) system [15].

The HSR is an interpretive Front of Pack Labelling system, first introduced in Australia and New Zealand in 2014, as a joint initiative between Government, public health, industry, and consumer groups. The system uses an algorithm to assign a star rating between 0.5–5 stars, and is intended to aid consumers in making healthier choices within categories [16,17]. The HSR algorithm rates foods on a per 100 g basis, considering both ‘negative nutrients’ (kilojoules, saturated fat, total sugars, and sodium), and ‘positive’ elements (fruit, vegetables, nuts and legumes, as well as protein and dietary fibre in some cases), which is then converted to a star rating [18]. Muesli bars were a key category of consideration in the ongoing HSR 5-year review, which noted they had received negative media attention based on products scoring “inappropriately high scores”, despite their categorisation as discretionary foods [19].

However, grain-based muesli bars may also be a potential source of positive ingredients and nutrients within the diet pattern, particularly considering whole grain and dietary fibre content, which are promoted within Australian Dietary Guidelines [12]. Widespread evidence supports whole grains and whole grain foods for their protective health benefits, including lower total and cause-specific mortality, type 2 diabetes [20,21,22,23,24], weight gain [25], and colorectal cancer [26]. Globally, low whole grain intake has been recognised as the second greatest dietary risk factor for mortality (behind sodium), and the greatest dietary risk factor for morbidity, responsible for more than 80 million Disability-Adjusted-Life-Years [27]. Irrespective of its well-documented health benefits, whole grain intake in Australia is low, with follow up data from the Australian Health Survey recording median intake for children at 16.5 g per day, and adults at 21.2 g/day—both less than half of the established Daily Target Intake (DTI) of 48 g per day for adults, and between 32 and 40 g per day for children [28,29,30]. Equally, a large body of evidence points to the benefits of dietary fibre and its role in reducing chronic disease risk, yet most Australians fall short, with more than half of children, and more than 70% of adults not meeting their respective targets [31].

Due to their popularity and increasing consumption in Australia, muesli bars are often criticised and met with confusion regarding their nutritional value, with a particular focus on sugar content. This study aimed to provide an overview of the nutritional status of grain-based muesli bars on shelf including muesli bars, grain-based bars, and oat slices in Australian supermarkets, and provide a comparison of 2019 with 2015 data.

## 2. Materials and Methods

An audit of grain-based muesli bars was conducted January 2019, in four major supermarkets in metropolitan Sydney (Aldi, Coles, IGA, and Woolworths). Collectively, these supermarket chains make up more than 80% of total Australian market share, and were chosen in preference to smaller, independent grocery stores in an attempt to reflect food choices that the majority of Australians are faced with during food shopping [32]. This recognised process has been outlined in previously published research [33] and the same process was utilised in the data collection from 2015. Smartphones were used to capture all information on food packaging, including ingredient lists, Nutrition Information Panels (NIP), health and nutrition claims, HSR, and any additional logos and endorsements. Outlined in Table 1 below, products accounted for in the audit included muesli bars, grain-based bars, (including fruit-filled bars and twists, and those made from wheat, puffed rice, or other grains), and oat slices. Products were further categorised to determine whether they were specifically marketed towards children, by the presence of cartoons, promotions, or sporting figures, as described in previous research [34,35]. Products excluded were fruit-based bars, fruit leather/straps, nutritional supplement bars (e.g. protein/‘low-carb’ bars), nut/seed based bars, and breakfast bars/biscuits (e.g. those designed as a meal replacement, indicated in the product name), in line with exclusions within the Healthy Food Partnership proposed reformulation targets [1]. A supplementary internet search was conducted through supermarket websites and identified manufacturer websites using key words such as “snack bars”, “muesli bars”, “grain-based bars”, “oat slices”, and “snack bars”, to ensure all products were captured.

Data from photographs taken at both timeframes (2015 and 2019) were transcribed into a Microsoft^®^ Excel^®^ spreadsheet (Version 2013, Redmond, Washington, DC, USA) for analysis. Information for the data entry included the NIP per serve and per 100 g, ingredients, percentage of whole grains, nutrition and health related claims, including whole grain, protein, dietary fibre, saturated fat, sugars, and sodium. Eligibility for products to make nutrition content claims was also assessed, in line with Food Standards Australia New Zealand and GLNCs Code of Practice for Whole Grain Ingredient Content Claims (The Code) [30], as well as proportion of products meeting the Healthy Food Partnership proposed reformulation targets for sugar reduction. HSR was not collected in 2015 as this was not on pack at this time. Where HSR was not featured on packaging, it was calculated for all products using the HSR website calculator [36]. A second, independent reviewer checked data for any inconsistencies and errors, and results were compared with 2015 data that followed the same process, to assess changes.

### Statistics

All data were checked for normality using Shapiro–Wilk test (IBM SPSS^®^, version 25.0, IBM Corp., Chicago, IL, USA) and mean and standard deviation were presented. As expected, there were missing values for dietary fibre and whole grain as these are often not presented unless a claim is being made on-pack, therefore dietary fibre and whole grain were analysed separately.

One-way ANOVA with post hoc Tukey analysis (IBM SPSS^®^, version 25.0, IBM Corp., Chicago, IL, USA) was used to compare differences per serve and per 100 g between (1) muesli bars, (2) grain-based bars, (including fruit-filled bars and twists, and those made from wheat, puffed rice, or other grains), and (3) oat slices for all available nutrients reported on-pack, including where relevant, dietary fibre, whole grain (g and %) and HSR (per 100 g). Independent samples *t*-test (IBM SPSS^®^, version 25.0, IBM Corp., Chicago, IL, USA) was used to compare whole grain and refined grain bars, which was defined according to each product’s eligibility for registration with The Code (≥8 g whole grain per manufacturer serve), a method that has been described in previously published research [37]. *T*-tests were also used to determine difference in HSR for all products /100 g, between whole grain and refined grain categories and for data per 100 g from 2015 compared with 2019.

## 3. Results

Data from 165 bars were collected, including 96 muesli bars, 46 grain-based bars, and 23 oat slices from 18 manufacturers where the top three (Nestle Ltd., Kellogg (Aust) Pty. Ltd. and Carman’s Fine Foods Pty. Ltd.), hold more than 60% market share (Retail World, December 2018) and have national distribution. Of these, 28 bars (17%) were identified as being specifically marketed towards children; these were predominantly grain-based bars (71%), with the remaining 8% muesli bars. Overall, mean serve size varied substantially between categories, with grain-based bars the smallest (27 g), followed by 35 g for muesli bars, and 55 g for oat slices.

There was a significant difference in nutrients including whole grain across all categories per serve and per 100 g (Table 2 and Table 3). Post hoc Tukey analysis (per serve) comparing muesli bars and grain-based bars revealed no significant differences in saturated fat (*p* = 0.181), carbohydrate (*p* = 0.365), sugars (*p* = 0.274), and sodium (*p* = 0.869). Grain-based bars and oat slices were significantly different across all nutrients and whole grain content. Conversely, muesli bars and oat slices were the closest in composition for dietary fibre and whole grain (*p* = 0.273 and *p* = 0.238 respectively) with grain-based bars significantly lower (*p* < 0.001). Almost all (95%) grain-based bars met the Australian Dietary Guidelines recommendations of 600 kJ or less as a ‘serve’ of discretionary food, as well as 61% of muesli bars, but only 8% of oat slices.

Comparing per 100 g, post hoc Tukey analysis revealed no difference in saturated fat (*p* = 0.558) between muesli bars and grain-based bars although all other nutrients and HSR were significantly different (*p* < 0.001). Similarly, all nutrients were significantly different between grain-based bars and oat slices except sodium (*p* = 0.952) and although muesli bars are most similar to oat slices in terms of dietary fibre and whole grain content as noted earlier, there were significant differences in fat (*p* = 0.001), saturated fat (*p* < 0.001), sodium (*p* = 0.009), and HSR (*p* = 0.001). Muesli bars were highest in dietary fibre, contributing an average of 9.4 g/100 g, the lowest in sodium (112.2 mg/100 g), and had a significantly higher HSR (3.0). They also contained the highest percentage of whole grain ingredients (40.7%) compared with grain-based bars and oat slices. The average HSR for all products was 2.7, but was higher for the 63% of products that displayed it on-pack (3.2 stars) compared to those that did not (1.8 stars).

The overall results for bars specifically targeted towards children were similar to the averages for grain-based bars, with an average of 1659 kJ ± 120 per 100 g, 6.1 ± 3.3g protein, 9.8 ± 3.5 g total fat, 4.3 ± 2.8 g saturated fat, 67 ± 7.9 g carbohydrate, 26 ±8.1 g sugars, 6.1 ± 4 g dietary fibre, and 161 ± 78.1 mg sodium. Children’s bars contained 19 ± 23.8% whole grain ingredients (contributing an average of 4.6 g to the 32–40 g Daily Intake Target for the 4–13 year old age group), and had an average HSR of 2.7 ± 1.1 stars, in line with the mean for the total snack bar category.

The percentage of products meeting nutrition claim criteria are presented in Table 4. More than half of muesli bars and oat slices were eligible for a ‘contains whole grain’ claim (compared to only 4% of grain-based bars), and 17% of oat slices were considered very high in whole grain. Six products did not report their percentage of whole grain ingredients, required to determine claim eligibility, so these were assumed as ineligible. Similar results were obtained for fibre claim eligibility, with 56% of the total category at least a source of fibre, mostly represented by muesli bars (69%), and oat slices (61%). The greatest proportion of grain-based bars were low in saturated fat (30%), compared to only 5% of muesli bars, and no oat slices. While none of the investigated bars were considered low in sugar, 48% overall met the most stringent proposed sugar reformulation target for muesli bars, (<25 g/100 g), and an additional 13% met the lower level proposed target of between 25–28 g sugar/100 g, with 29% falling outside the criteria.

As outlined in Table 5, bars categorised as whole grain (≥8 g per manufacturer serve) were significantly higher in energy, total fat, and dietary fibre, and lower in sugars and sodium than refined grain bars. Interestingly, there was no significant difference noted in HSR between whole grain and refined grain bars, with 0.7 star between those categorised as whole grain and the remaining ‘non-whole grain bars’ which were categorised as refined grain bars.

In regards to other on-pack claims, ‘No artificial colours/flavours/preservatives’ was the most common claim made on packaging, featuring on almost three-quarters (73%) of the total category, and on 91% of oat slices, 80% of grain-based bars, and 66% of muesli bars. More than half made a dietary fibre claim (56%), including 60% of both oat slices and muesli bars, and 30% of grain-based bars. Similarly, 49% made a whole grain claim on-pack, mainly seen on oat slices (70%), and muesli bars (68%), with only 9% of grain-based bars making this claim. An additional 28 products were eligible, but did not make a whole grain claim.

Compared with 2015 (Table 6), 3.5% fewer bars were captured (171 versus 165), with apparent growth in the number of muesli bars (82 to 96 products), and oat slices (18 to 23 products), but a decline in grain-based bars (71 to 46 products), these being the most nutritionally poor products within the category. Over time, there was a significant decrease in total sugars from 26.6 g/100 g to 23.7 g/100 g (*p* < 0.001) across the total category in the four years since 2015, largely attributed to muesli bars, containing 4.2 g/100 g less sugars, while grain-based bars remained stable, and oat slices decreased by 1.1 g/100 g. The proportion of whole grain bars within the category increased, from 35 to 66% in four years (60/171 up to 109/165 bars). HSR data was not captured in 2015 due to the system being newly introduced, so no comparison of this metric over time was possible.

## 4. Discussion

Despite their widespread popularity, consumption of grain-based muesli bars are discouraged by the Australian Dietary Guidelines based on their classification as a discretionary food. This study aimed to provide a comprehensive overview of the nutritional status of grain-based muesli bars on shelf in Australian supermarkets, compared to data collected in 2015.

Overall, wide nutrient ranges were demonstrated between and within the categories examined although muesli bars are treated as a homogenous category in food policy and in advice to consumers. A major factor influencing these differences was the range in average serve sizes, with oat slices more than double that of grain-based bars. Serve size discrepancy may be a point of confusion for shoppers, as nutrient content of the smaller sized grain-based bars may appear more favourable, yet these were the highest in some nutrients of concern on a per 100 g basis. Conversely, oat slices are larger and appear the highest in some positive nutrients per serve, but not when compared per 100 g. This may suggest that the nutrition features of bars may be difficult to compare using the per serve nutrition information at the supermarket shelf. This has been previously described as ‘health framing’, whereby the impression of a healthier product may lead to overconsumption, however as all bars examined were individually wrapped and therefore portion controlled, this may be less of a concern than in other snack food categories such as cakes and biscuits. These findings are consistent with prior research in Australia which found significant variability in manufacturer serve size within both discretionary [38,39], and core food groups [40,41], and are partly explained by the lack of regulation around standard serving sizes in Australia, which is determined by food manufacturers [40].

Differing ingredients were also a major factor influencing variations in nutrition profile and serve size. Many grain-based bars consisted of puffed or flaked grains (such as corn or rice), which were likely lighter in weight than whole grains, more commonly found in muesli bars and oat slices. Oat slices often contained butter and coconut, both known for their high levels of saturated fat. Additionally, muesli bars and oat slices were all based on oats, which are unique among grains for their higher fat content (6–8%, compared to 2–3% in other grains [42]). The difference in ingredients provides basis for considering further differentiation within this category and at the same time, questions the broad categorisation of ‘muesli bars’ within the discretionary food group.

Almost one in five bars (17%) in 2019 were specifically marketed towards children, and these were mainly within the grain-based bars category (which are smaller and often made with puffed grains). Generally, these were less nutritious options, being lower in protein, dietary fibre, and whole grain, and higher in sugar than the category on average. Previous research has echoed this finding, with the products designed to appeal to children generally higher in some negative nutrients [34]. Encouragingly, their nutritional value was reflected in the average HSR of less than 3 stars, which has been determined as a cut off point for consumers identifying a food as unhealthy [43].

‘Snackification’, or the demand for convenience foods to suit modern lifestyles may drive continued innovation and reformulation. New Nutrition Business identified snacking as a key driver of food choice in 2018 and 2019, pointing to examples of manufacturers reinventing foods that were once impossible to eat on-the-go, such as peanut butter in portioned sachets and microwave porridge in individual pots, possibly increasing market competition for muesli bars as traditional snack foods [44]. When considering the top three contributors to adults (19–70+ years) discretionary food intake, the Australian Institute of Health & Welfare’s 2018 Nutrition Across the Life Stages report listed alcohol, cakes/muffins/pastries, and soft drinks [45]. Similarly, a 2017 review analysing Australian children’s discretionary food intake identified cakes/muffins/slices (4.2%), sweet biscuits (2.9%), and potato crisps/similar snacks (2.7%) as the top contributors to total energy, and the greatest contributors to added sugar were sugar-sweetened soft drinks (18.6%), cakes, muffins, and slices (10.6%), and cordials (6.7%). Conversely, ‘sweet snack bars’ (which included muesli/cereal bars, and fruit/nut/seed bars) contributed only 1.2% to total energy, and 1.6% added sugars [46]. When this is considered in the context of a typical Australian school lunchbox, including “about one sandwich, two biscuits, a piece of fruit, a snack of either a muesli/fruit bar or some other packaged snack, and a drink of fruit juice/cordial or water” [4], the particular focus on muesli bars as a food of concern may need to be reassessed against the full range of options that could be included in this meal occasion. Discretionary foods such as biscuits, cakes, potato chips, and cordial offer minimal nutritional benefits, so encouraging healthier options within the muesli bar category, alongside core foods in preference to these may be more beneficial advice to consumers and parents who are already under pressure to provide convenient, nutritious snacks.

Comparisons with 2015 data (in Table 6) are suggestive of improvements in terms of added sugars and whole grain content made by food industry. Reformulation aims to improve the nutritional content of manufactured foods, either by increasing beneficial nutrients, or reducing risk-associated nutrients. Often, manufacturers make modest nutritional changes over a period of time to allow consumers’ tastes to adjust accordingly, referred to as “health by stealth” [47], but in recent years Australian muesli bar manufacturers have openly shared efforts to reduce salt, fat, sugar, and increase dietary fibre [48]. There is evidence to show reduction targets are effective, with a 2018 review of voluntary sodium reduction targets in soup demonstrating a 6% reduction in sodium levels in soup products between 2011 and 2014, with 67–74% of products compliant with targets [49]. Similarly, Australia’s National Heart Foundation has reported significant reductions in line with targets set by the Food and Health Dialogue, such as 10% less sodium in bread and processed meats, and 32% less sodium in breakfast cereals [50], indicating that proposed targets set by the Healthy Food Partnership may encourage further improvements in the added sugars content of muesli bars.

Authors of the 2017 Global Burden of Disease study speculated that dietary policies focused on promoting consumption of whole grains, fruits. and vegetables, and other core food groups may have a greater effect than policies targeting excess consumption of sugar and fat [27]. Within the current study, whole grain bars were clearly identified as a healthier option overall, providing more protective nutrients, and fewer negative nutrients than refined grain bars. Across categories, the majority of oat slices and muesli bars were whole grain (≥8 g per manufacturer serve), and provided the equivalent of at least 30% of an adult’s 48 g Daily Target Intake for whole grain, and up to half of a child’s daily whole grain requirement (32–40 g/day) [30]. In light of this, whole grain bars may present a convenient, portion controlled, and accepted vehicle for whole grain, and their consumption over refined grain bars could aid in bridging the significant gap in consumption. Unlike other nutrients, whole grain claims are not regulated by Food Standards Australia New Zealand, but are instead encouraged through GLNCs voluntary Code of Practice for Whole Grain Ingredient Content Claims (The Code), introduced in Australia and New Zealand in 2013 to encourage evidence-based promotion of whole grain foods. GLNC utilises audits of grain-based foods to monitor the operation of The Code and provide feedback to industry as necessary. While 60% of eligible bars were registered with The Code, its voluntary nature, and the fact that the percentage of whole grain ingredients is not mandatory in the ingredients list means deciphering which are whole grain options is not always clear to consumers. This was highlighted by the six bars identified that contained whole grain ingredients (such as rolled oats, and whole grain wheat), but did not report their percentages, so it was unclear whether they met The Code’s whole grain criteria. Encouragingly, the number of whole grain bars have increased by 31% since 2015, suggesting positive changes have been made by manufacturers to existing products, new whole grain products have been added to the market due to consumer demand, or that labelling has been updated to more clearly communicate whole grain content.

The variability in nutrients supplied within the grain-based muesli bar category, combined with their popularity, may point towards education as the more powerful tool in supporting consumers to choose healthier products, in preference to discouraging consumption. The concept of ‘knowledge-is-power’ has been explored in previous research, with a review from the USA determining consumers with greater nutrition knowledge were more likely to consult nutrition labels, which may lead to healthier food choices [51]. The HSR attempts to clarify complex nutrition information and arm consumers with the knowledge to make healthier choices within food categories, and has been shown to perform well in directing consumers towards healthier, higher-scoring foods [43,52,53]. HSR scores for the bars category ranged from 1–5 stars, yet there was no significant difference between refined and whole grain varieties, with only 0.7 of a star between products. This finding highlights a shortcoming of the algorithm used to assign products a star rating, and builds on previous research that demonstrated an inability to differentiate whole grain and refined grain breads, breakfast cereals, rice, and flour products, as it does not directly account for, or reward foods for whole grain content [37]. There is a clear opportunity to refine the HSR by recognising whole grain as a positive food component, which could play a role in discerning healthier food choices across numerous categories, including muesli bars. However, to meet its objective of simple nutrition comparisons within categories, widespread uptake of a voluntary front-of-pack labelling system such as the HSR is required. Almost two-thirds (63%) of bars examined displayed a HSR, comparatively higher than overall uptake, which is estimated at 28% [16]. Consistent with existing literature, bars displaying a HSR tended to have higher scores, suggesting the system may be used strategically within and across brands [16,54]. Conversely, industry appear to be using the HSR as an incentive to improve a product’s nutritional value, with recent studies in Australia and New Zealand identifying upwards of 83% of products displaying a HSR had been reformulated to increase their score [54,55].

Strengths of this study include its comprehensive nature, and to our knowledge, it is the first study that has reviewed muesli bars on shelf in Australia, with a comparison made to previously collected data. Also, where HSR was not provided, we calculated this for a more accurate representation of HSR across the category. However, there were some limitations. The research was focused only on grain-based bars, excluding others—such as nut bars and protein and low-carb bars—which may also be consumed as snacks though to a lesser extent than muesli bars [56]. While all efforts were made to capture the category in its entirety, differences may exist between geographic areas. As previously stated, reporting of dietary fibre and whole grain within the ingredients and Nutrition Information Panel is not mandatory in the absence of an on-pack claim, so was not always declared, and thus there was some missing data. Finally, we did not conduct an independent nutrition analysis, and were reliant on manufacturer information.

## 5. Conclusions

Although categorised as discretionary, there are significant nutrient differences across grain-based muesli bars, with well-chosen bars providing valuable amounts of whole grain and dietary fibre. Muesli bars are a widely consumed snack food, particularly among younger age groups in Australia, yet their contribution and role in the diet is controversial, based on their classification at discretionary by Australian Dietary Guidelines. This study demonstrated significant variation between and within the category, with the whole grain options emerging as more nutritious compared to refined grain bars, and an indication of sugar reduction since 2015. Within a balanced diet, it is clear that some muesli bars can offer a convenient and nutritious snack, with many bars providing around 30% of an adult’s, and up to half of a child’s daily requirement for whole grain, and more than half of all products are at least a source of fibre. Both whole grains and dietary fibre are encouraged within Dietary Guidelines yet intakes across age groups tend to fall short of dietary targets. The current HSR algorithm does not appear to be overly favouring muesli bars (with an overall score of 2.7), and instead, could be improved to capture and differentiate whole grain options. Ongoing promotion of the higher HSR scoring bars, alongside proposed voluntary sugar reformulation targets and trends such as snackification, may be suggestive of opportunities and incentives for manufacturers to further improve the current range of products. Clearer classification within policy initiatives utilising evidence-based assessment of available products may help refine advice from healthcare professionals, and may be key in providing better direction for consumers to make healthier and acceptable snack food and lunchbox choices.

## Figures and Tables

**Table 1:** Classification of categories.

Category	Description
Muesli bar	Baked or cold-formed bars where oats made up ≥5% of the product OR were one of the first five ingredients listed on the Nutrition Information Panel (NIP)
Grain-based bar	Baked or cold-formed bars where grain ingredient (s) (excluding oats) made up ≥5% of the product OR grains (excluding oats) were one of the first five ingredients listed on the NIP
Oat slice	Soft-baked bars with the word ‘slice’ in the product name, where oats made up ≥5% of the product OR were one of the first five ingredients listed on the NIP

**Table 2:** Nutrients per serve (mean & SD): muesli bars, grain-based bars, and oat slices including whole grain.

Nutrient Criteria	Muesli Bars (*n* = 96)	Grain-Based Bars (*n* = 46)	Oat Slices (*n* = 23)	*p*-Value	Total Bars (*n* = 165)
Serve Size (g)	35 ± 7.5	27 ± 7.0	55 ± 29.9	<0.001	35 ± 15.7
Energy (kJ)	614.4 ± 155.6	428.4 ± 91.6	1007.7 ± 565.6	<0.001	617.4 ± 301.0
Protein (g)	3.1 ± 1.7	1.5 ± 0.6	4.1 ± 2.5	<0.001	2.8 ± 1.9
Fat (g)	5.6 ± 2.9	2.3 ± 1.4	11.2 ± 7.0	<0.001	5.4 ± 4.4
Saturated Fat (g)	1.7 ± 1.2	1.1 ± 0.7	7.1 ± 4.3	<0.001	2.3 ± 2.7
Carbohydrate (g)	19.7 ± 3.9	18.0 ± 5.0	29.6 ± 15.8	<0.001	20.6 ± 7.9
Sugars (g)	7.3 ± 2.5	8.2 ± 3.4	12.0 ± 5.3	<0.001	8.2 ± 3.6
Dietary Fibre (g)	3.2 ± 1.6	1.6 ± 1.5	3.8 ± 2.4	<0.001	2.8 ± 1.9
Sodium (mg)	39.7 ± 44.1	43.5 ± 19.9	99.0 ± 61.4	<0.001	49.0 ± 46.4
Whole Grain (g)	14.2 ± 4.8	1.0 ± 2.8	16.1 ± 8.2	<0.001	10.7 ± 7.9

One Way ANOVA 95% CI.

**Table 3:** Nutrients, whole grain, and HSR/100 g (mean & SD) in muesli bars, grain-based bars, and oat slices.

Nutrient Criteria	Muesli Bars (*n* = 96)	Grain-Based Bars (*n* = 46)	Oat Slices (*n* = 23)	*p*-Value
Energy (kJ)	1770.9 ± 180.3	1633.4 ± 188.2	1817.9 ± 112.7	<0.001
Protein (g)	8.6 ± 3.2	5.4 ± 1.9	7.3 ± 1.0	<0.001
Fat (g)	15.4 ± 5.1	9.1 ± 5.8	20.0 ± 4.4	<0.001
Saturated fat (g)	5.0 ± 3.3	4.3 ± 3.7	12.9 ± 3.3	<0.001
Carbohydrate (g)	56.7 ± 8.3	67.6 ± 7.4	53.9 ± 3.9	<0.001
Sugars (g)	20.9 ± 5.6	29.8 ± 6.4	23.1 ± 4.3	<0.001
Dietary Fibre (g)	9.4 ± 5.1	5.8 ± 6.1	6.6 ± 0.8	<0.001
Sodium (mg)	112.2 ± 121.0	166.7 ± 69.9	174.5 ± 46.7	0.002
HSR	3.1 ± 1.1	2.4 ± 1.0	1.8 ± 0.4	<0.001
% Whole Grain	40.7 ± 11.8	4.8 ± 13.4	29.7 ± 4.6	<0.001

One Way ANOVA 95% CI.

**Table 4:** Percentage of products meeting claim criteria and proposed reformulation targets *.

	Muesli Bars (*n* = 96)	Grain-Based Bars (*n* = 46)	Oat Slices (*n* = 23)	Total Snack Bars (*n* = 165)
Eligible for WG claim (≥8 g/manufacturer serve)	90	4	91	66
Contains WG (≥8 g/manufacturer serve)	58	4	65	43
High in WG (16–24 g/manufacturer serve)	41	0	4	16
Very High in WG (≥24 g/manufacturer serve)	4	0	17	6
Source of Fibre (≥2–<4 g/serve)	69	26	61	56
Good Source of Fibre (≥4–<7 g/serve)	5	2	4	4
Excellent Source of Fibre (≥7 g/serve)	5	2	22	7
Low in Saturated Fat (≤1.5 g/100 g)	5	30	0	12
Low in Sugar (≤5 g/100 g)	0	0	0	0
Meets Proposed Sugar Reformulation Target 25–28 g/100 g *	9	11	4	9
Meets Proposed Sugar Reformulation Target <25 g/100 g *	78	24	65	61

* Healthy Food Partnership proposed reformulation targets (September 2018).

**Table 5:** Whole grain versus refined grain nutrients (per 100 g) (mean and SD).

NIP	Whole Grain * (*n* = 109)	Refined Grain ** (*n* = 56)	*p*-Value
Energy (kJ)	1772.6 ± 171.1	1673.8 ± 199.6	0.044
Protein (g)	8.4 ± 2.9	5.9 ± 2.3	0.384
Fat (g)	16.1 ± 5.2	10.9 ± 7.0	0.034
Saturated Fat (g)	6.2 ± 4.4	5.3 ± 4.3	0.389
Carbohydrate (g)	56.2 ± 7.4	65.4 ± 9.3	0.059
Sugars (g)	20.9 ± 5.1	29.1 ± 6.6	0.043
Dietary Fibre (g)	8.6 ± 4.2	6.6 ± 6.7	0.008
Sodium (mg)	119.5 ± 111.0	168.4 ± 82.2	0.005
HSR	3.1 ± 1.1	2.4 ± 1.0	0.075

Independent samples *t*-test 95% CI. * Based on eligibility for registration with GLNCs Code of Practice for Whole Grain Ingredient Content Claims (≥8 g per manufacturer serve). ** Includes six bars that did not report percentage of whole grain ingredients.

**Table 6:** Comparison of nutrients and whole grain in total bars between 2015 and 2019 per 100g (mean and SD).

Nutrient Criteria	Total Bars 2015 (*n* = 171)	Total Bars 2019 (*n* = 165)	*p*-Value
Energy (kJ)	1700 ± 179.9	1739.1 ± 186.7	0.049
Protein (g)	6.6 ± 2.1	7.5 ± 3.0	0.001
Fat (g)	13.1 ± 6.2	14.1 ± 6.2	0.089
Saturated Fat (g)	5.7 ± 4.4	5.9 ± 4.4	0.610
Carbohydrate (g)	62.3 ± 8.4	59.3 ± 9.2	0.002
Sugars (g)	26.6 ± 7.2	23.7 ± 6.8	<0.001
Dietary Fibre (g)	6.6 ± 4.1	7.9 ± 5.2	0.203
Sodium (mg)	143.1 ± 104.5	136.1 ± 104.5	0.540
% Whole Grain	30.0 ± 15.0	38.8 ± 11.2	0.009

Independent samples *t*-test 95% CI.
